# Comparing the Perceived Realism and Adequacy of Venipuncture Training on an in-House Developed 3D-Printed Arm With a Commercially Available Arm: Randomized, Single-Blind, Cross-Over Study

**DOI:** 10.2196/71139

**Published:** 2025-11-04

**Authors:** Susan Gijsbertje Brouwer de Koning, Amy Hofman, Sonja Gerber, Vera Lagerburg, Michelle van den Boorn

**Affiliations:** 1 3D Lab, Department of Computerization, Automation and Medical Technology (iMED), OLVG Hospital, 9 Oosterpark, Amsterdam, 1091 AC, The Netherlands, 31 020 599 91 11; 2 Department of Research and Epidemiology, OLVG Hospital, Amsterdam, The Netherlands; 3 Skillslab, OLVG Hospital, Amsterdam, The Netherlands; 4 Department of Medical Physics, OLVG Hospital, Amsterdam, The Netherlands; 5 Department of Medical Physics and Instrumentation, St. Antonius Ziekenhuis, Nieuwegein, The Netherlands

**Keywords:** venipuncture, phlebotomy, blood draw, 3D printing, vascular phantom

## Abstract

**Background:**

The venipuncture is one of the most frequently performed procedures in health care. Arm phantoms are available for training, because the procedure itself can be challenging. These phantom arms do not represent a realistic setting and do not offer opportunities to train challenging scenarios.

**Objective:**

This randomized, single-blind study aimed to train health care workers on both a commercially available injection arm and an in-house developed 3D-printed arm, to evaluate the perceived realism and adequacy of training on both arms.

**Methods:**

Participants were trained on both the commercially available arm (arm A) and the 3D-printed arm (arm B). Participants were randomized and blinded from knowing which arm they started training on. A questionnaire was filled in on, among others, the perceived realism of the arm (0 for not realistic, 100 for realistic) and adequacy of the training (inadequate, moderate, or adequate).

**Results:**

A total of 68 participants evaluated the perceived realism of arm A and B, which were scored on average 62.97 (SD 21.47) and 63.79 (SD 17.45), respectively. The difference in perceived realism of the two arms was not statistically significant (based on the paired *t* test, mean difference=−0.82, *P*=.78). Training on arm A was reported inadequate by 7% (5/68 participants), moderately adequate by 31% (21/68), and adequate by 62% (42/68). This was not significantly different from arm B (marginal homogeneity test, *P*=.74), with 4% (3/68), 38% (26/68), and 57% (39/68), respectively, reporting that the training was inadequate, moderately adequate, and adequate.

**Conclusions:**

The 3D-printed arm is as realistic and provides an equally adequate training compared to the commercially available arm. The 3D-printed arm offers the additional possibility to design different models representing several levels of difficulty for vascular morphology. This potentially lowers the number of venipuncture failures by preparing health care workers on challenging scenarios.

## Introduction

The venipuncture (or phlebotomy, blood draw) is one of the most frequently performed procedures in hospitals and is predominantly carried out for diagnostic purposes [1] Although the venipuncture is recognized as a safe and relatively easy procedure, it can be a challenging procedure especially in certain groups, for example, in patients with obesity, patients with a dark skin, or patients undergoing dialysis or chemotherapy. Minor complications include bruising and hematoma and occur in about 12% of venipunctures, while serious complications such as diaphoresis with hypotension or syncope (vasovagal) occur in 3% of patients [2]. There is a minor chance of nerve injury (1/67,000 venipunctures) and also phlebitis is rarely reported [3,4].

The risk of complications is enhanced in case of failure of the venipuncture, that is, a failed needle insertion where no blood samples can be collected, and a second attempt of venipuncture is required. It has been shown that in order to achieve a rate of successful venipunctures close to 99% at the first attempt, one year of practice is necessary [5]. When two attempts both fail for blood sampling, the protocol of most hospitals recommends contacting the anesthesiology department to perform the venipuncture procedure instead. In 2023, this happened 12.233 times at the Onze Lieve Vrouwe Gasthuis (OLVG), a large city hospital in Amsterdam (unpublished data, Business Intelligence Department, OLVG, 2023).

In order to lower this number, proper training and supportive supervision of health care professionals learning the venipuncture is fundamental and therefore recommended by the World Health Organization guidelines on drawing blood [6]. Medical education has evolved from the traditional apprenticeship training to more sophisticated training methods, such as training on simulators (venipuncture arm phantoms) and the use of e-learning modules for theoretical education and electronic simulation [7]. Simulation training can help to improve the venipuncture technique [8]. In addition, training can also improve self-confidence, which also might decrease the number of venipuncture failures [9].

To learn the venipuncture procedure, the current practice is a training on a commercially available arm phantom. This is a phantom of an arm with vessels filled with a liquid that simulates blood. The vessels are embedded in a firm material to enable localization of the vessels by palpation through a layer of skin. Although this arm is a major advancement to practice the venipuncture routine in a safe learning environment yet, there is room to improve training possibilities of the injection arm. Disadvantages of the current training arm are both the realism of the arm and some practical issues.

3D-printing gives the possibility to design and print tailor-made arms. This offers the opportunity to design different versions or models representing several levels of difficulty for vascular morphology to train both novice and experienced health care workers. As a first step towards these advanced tailor-made arms, a regular 3D arm phantom. The aim of this study was to compare the perceived realism and the reported adequacy of the training on a commercially available injection arm to a 3D printed arm.

## Methods

### Study Design

In this randomized, single-blind cross-over comparison study, participants were recruited among the hospital staff of OLVG, a large city hospital in Amsterdam. Information about the study was given via internal communication channels, that is, the intranet and digital newsletter. Participants could apply for the study voluntarily (voluntary response sampling). Requirements for the application were an age of ≥18 years and a medical license to perform peripheral intravenous injections.

All training sessions were supervised by the same trainer. The training started with a short introduction to assess the participant’s initial experience and knowledge. A short explanation of the procedure was given, and participants were asked for their learning question. After this, the procedure was demonstrated by the trainer on a training arm. This arm was not part of this study. Participants could practice as often as they needed; the trainer observed and provided feedback on their actions where needed.

**Figure 1.**
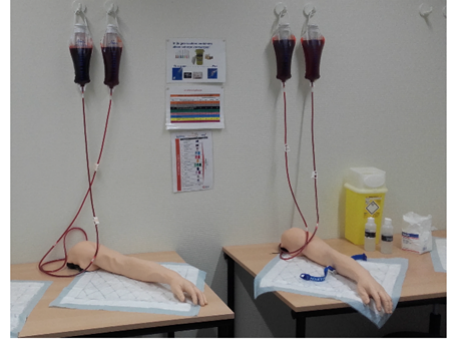
Commercially available venipuncture arm phantom (arm A) and the 3D-printed arm (arm B) during the training.

**Figure 2.**
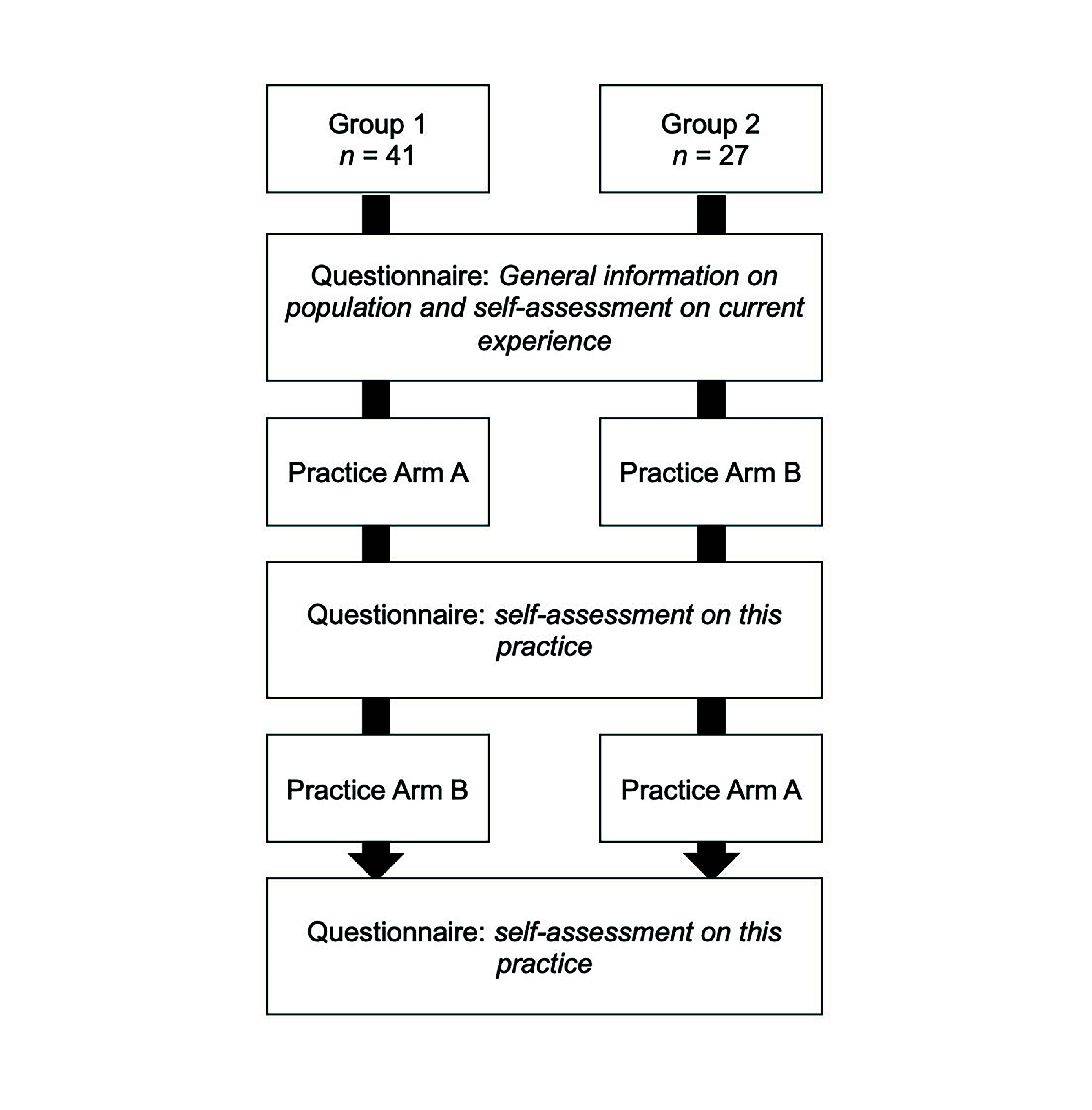
Sequence of training and questionnaires.

Participants were randomized into two groups: group 1 started the training on the commercially available injection arm (arm A) and continued with training on the 3D-printed arm (arm B), while group 2 started the training on arm B and continued training on arm A (Figure 1). The participants received a closed envelope containing the information on which arm to start the training on (A or B). Participants were blinded from knowing which arm was the commercially available arm, and which arm was the 3D-printed arm, as the same sleeves were used for arm A and arm B. Before and after training on each arm, participants were asked to fill in a questionnaire (Figure 2).

### Ethical Considerations

Participants had to give informed consent to participate in the study. Participants were able to opt out. No compensation was provided to any participant. The study was approved by the local ethics committee of the OLVG, the Advisory Committee on Scientific Research (approval: WO19.065), and data were collected anonymously.

### Measurements

The primary outcome measures were assessed using a questionnaire after each training round: perceived realism of the arm (rated on a scale from 0 [not realistic] to 100 [realistic]) and adequacy/quality of the training (three response categories: inadequate, moderate, or adequate). In addition, information about the participants (eg, role, department, or experience with venipuncture) was collected using these questionnaires (Multimedia Appendix 1).

### Sample Size

No published data were available on ratings of realism or training adequacy for phantom arms. Therefore, assumptions were made based on expert and user opinions. For the commercially available arm, a mean realism score of 70 (on a 0‐100 scale) was assumed, together with a conservatively assumed standard deviation of 20. To detect a mean difference of 10 points with 80% power at a two-sided alpha level of .05, a sample size of 65 participants was required.

### Arm Phantoms

The commercially available arm phantom is designed to train venipuncture and intravenous infusion (NASCO Healthcare). Veins are accessible at the antecubital fossa, along the forearm, and at the back of the hand. There are visible veins, as well as invisible veins that can only be determined by palpation. Artificial blood can be drawn from the vessels (NASCO Healthcare, Life/form®). The arm is designed with a skin that has a soft touch when palpating the vessels.

**Figure 3.**
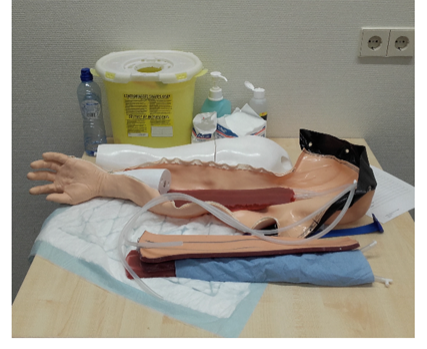
The 3D printed arm.

The commercially available arm phantom for venipuncture was the basis of the design of the tailor-made 3D-printed arm: a digital 3D model of the commercially available arm was acquired through a 3D surface scan (Artec EVA 3D scanner, Artec Studio 15 Software). The digital 3D model was edited in Meshmixer (Autodesk, Inc.) and prepared for printing in the program Simplify3D. The arm was printed from glycol modified polyethylene terephthalate using the Ultimaker printer (Ultimaker BV). Silicone tubes were used to simulate the vessels (Dispomed Ltd; Figure 3). The skin of the arm was similar to the skin of the commercially available skin. This was the first time a 3D-printed arm was produced by our laboratory. There was no pilot performed on this arm in advance of using the arm for the training with participants.  

### Statistical Analysis

The study population was characterized using descriptive statistics. Continuous variables were presented as mean (SD) or median (IQR), depending on normality (assessed by visual inspection of histograms and Q-Q plots). Categorical variables were presented as frequencies and percentages (n, %).

As the primary outcomes were rated twice by the same participants (cross-over design), paired tests were applied. The difference in perceived realism of the arm was evaluated using a paired samples *t* test. The difference in adequacy ratings of the training was evaluated using a marginal homogeneity test for related samples.

To explore potential sequence effects (order of training, determined by randomization), results were visualized per randomization group, and perceived realism scores were compared between groups using independent sample *t* tests.

All tests were two-sided; *P* values below .05 were considered statistically significant. Analyses were performed using SPSS version 27 (IBM).

## Results

### Study Population

Characteristics of group 1 and group 2 are presented in Table 1. Most participants in both groups were nurses or physician assistants (Group 1 30/41, 73%; Group 2 15/27, 56%)). The median number of years of experience with the venipuncture was 1 year for both groups (IQR 0‐4 and 0‐3, for groups 1 and 2, respectively). Group 1 rated their competence in venipuncture on a 0‐100 scale with a median of 50 (IQR 18‐65), while group 2 rated their competence with a median of 40 (IQR 20‐70). The self-rate of successful venipunctures at first attempt was approximately equal between the groups, with a median of 40 on a 0‐100 scale. Half of the total population (34/68, 50%) performed a venipuncture daily over the last 6 months. Almost all participants (57/68, 83.8%) had a venipuncture training before, mostly on phantom arms.

**Table 1.** Characteristics and self-reported experience of study population.

Characteristic	Total population (N=68)	Group 1 (N=41)	Group 2 (N=27)
Role, n (%)			
Nurse or nurse specialist	45 (66.2)	30 (73.2)	15 (55.6)
Nurse in training	5 (7.4)	3 (7.3)	2 (7.4)
Medical doctor	3 (4.4)	1 (2.4)	2 (7.4)
Medical intern	3 (4.4)	1 (2.4)	2 (7.4)
Other^a^	12 (17.6)	6 (14.6)	6 (22.2)
Department, n (%)			
Surgery	6 (8.8)	6 (14.6)	0 (0.0)
Geriatrics	12 (17.6)	5 (12.2)	7 (25.9)
Gynecology	14 (20.6)	7 (17.1)	7 (25.9)
Intensive Care	5 (7.4)	4 (9.8)	1 (3.7)
Lung	2 (2.9)	2 (4.9)	0 (0.0)
Gastroenterology	3 (4.4)	2 (4.9)	1 (3.7)
Neurology	4 (5.9)	2 (4.9)	2 (7.4)
Oncology	3 (4.4)	2 (4.9)	1 (3.7)
Orthopedics	5 (7.4)	3 (7.3)	2 (7.4)
Emergency room	2 (2.9)	2 (4.9)	0 (0.0)
Other^b^	12 (17.6)	6 (14.6)	6 (22.2)
Mean number of years of experience with venipuncture, median (IQR)	1 (0‐4)	1 (0‐4)	1 (0‐3)
Self-rating competence in venipuncturing (0‐100), median (IQR)	50 (20‐70)	50 (18‐65)	40 (20‐70)
Self-rating of successful venipunctures at first attempt (0‐100), median (IQR)	40 (0‐70)	40 (1‐70)	40 (0‐60)
Number of venipunctures within the last six months, n (%)			
Once a month or less	15 (22.1)	12 (29.3)	3 (11.1)
A few times a month	7 (10.3)	5 (12.2)	2 (7.4)
A few times a week	12 (17.6)	6 (14.6)	6 (22.2)
On a daily basis	34 (50.0)	18 (43.9)	16 (59.3)
Number of failed attempts and getting help over the last month, n (%)		0 (0‐2)	1 (0‐2)
I have had a venipuncture training before	57 (83.8)	33 (80.5)	24 (88.9)
I have had a training on a commercial venipuncture arm phantoms before	39 (57.4)	23 (56.1)	16 (59.3)

a Other: students medicine/technical medicine, obstetrician, dialysis assistant, radiologist.

b Other: radiology, ophthalmology, medical technology, internal medicine, otorhinolaryngology, psychiatry.

### Perceived Realism of the Commercial Venipuncture Arm Phantom and the 3D-Printed Arm Phantom

The perceived realism of arms A and B in the total population were on average 62.97 (SD 21.47) and 63.79 (SD 17.45), respectively. There was no statistically significant difference between the reported perceived realism of arm A and B (mean difference −0.82, 95% CI −6.80 to 5.16, *P*=.78).

### Adequacy of the Training With Commercial Venipuncture Arm Phantom and the 3D-Printed Arm Phantom

Training on arm A was reported inadequate by 5/68 (7%) of the participants, moderately adequate by 21/68 (31%), and adequate by 42/68 (62%). This was not significantly different from arm B (*P*=.74), with 4% (3 out of 68), 38% (26 out of 69) and 57% (39 out of 68), respectively, reporting that the training was inadequate, moderately adequate, and adequate.

### Perceived Realism Per Randomized Group

**Figure 4.**
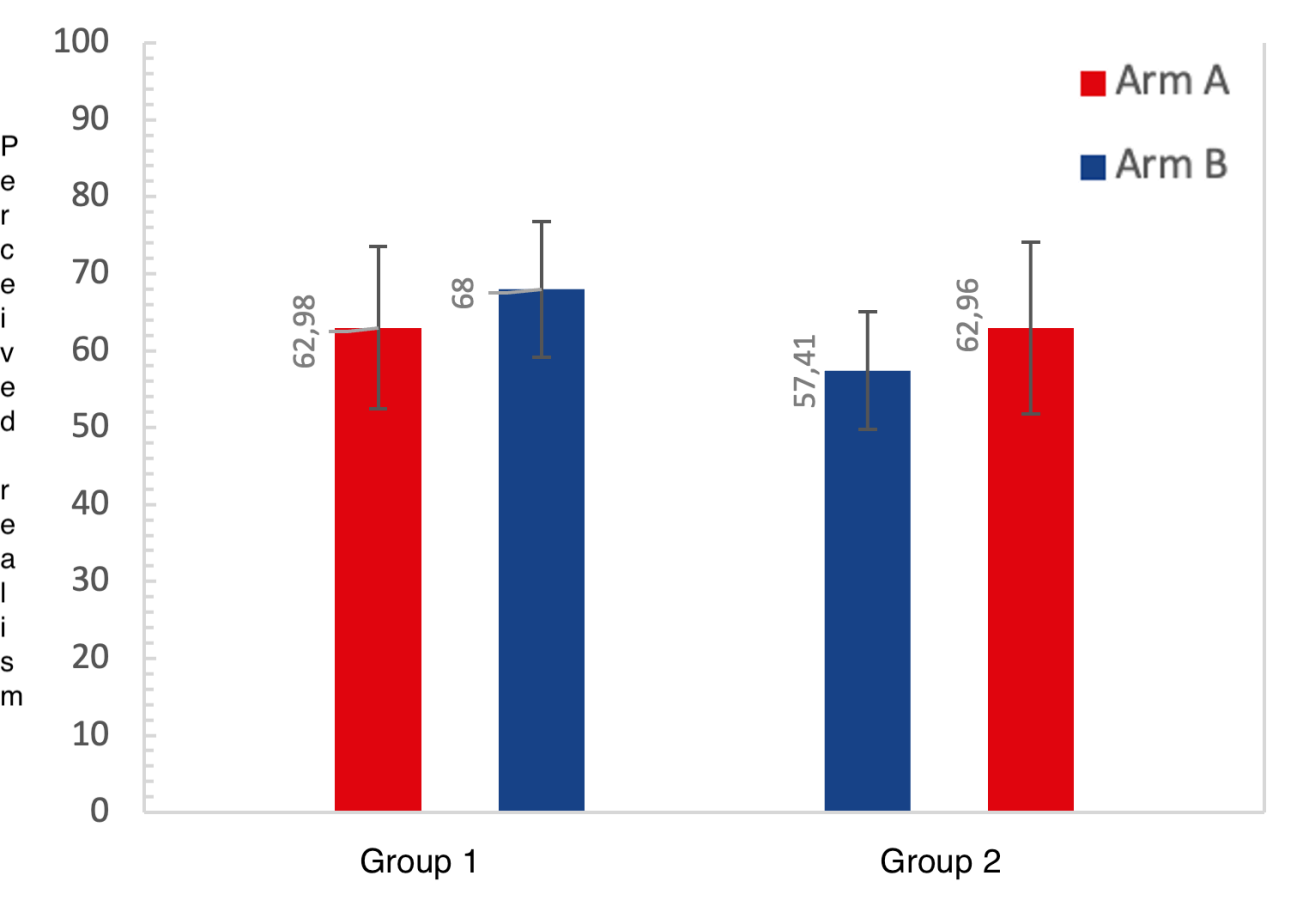
The perceived realism of arm A (commercially available venipuncture arm phantom) and B (3D-printed arm phantom) (0 for not realistic, 100 for realistic).

To explore whether training sequence influenced the reporting of perceived realism, Figure 4 shows perceived realism scores stratified by randomized groups. For the commercially available arm (arm A), perceived realism did not differ between groups (mean difference 0.01, 95% CI −10.69 to 10.71, *P=*.998). In contrast, the perceived realism of the 3D-printed arm phantom (arm B) was reported to be significantly lower in group 2, who started training on this arm (mean difference 10.59, 95% CI 2.29 to 18.89, *P*=.013).

## Discussion

### Principal Findings

The objectives of this study were to train health care workers on both a commercially available injection arm and an in-house developed 3D-printed arm and to evaluate the perceived realism and adequacy of training on both arm phantoms. Our results indicate that the 3D-printed arm phantom is as realistic and provides an equally adequate training compared to the commercially available arm phantom. The perceived realism of the commercially available arm phantom and the 3D-printed arm phantom were on average 62.97 (out of 100, SD 21.47) and 63.79 (SD 17.45), respectively (*P*=.784). Training on the commercially available arm was reported inadequate by 5/68 (7%) of the participants, moderately adequate by 21/68 (31%), and adequate by 42/68 (62%). This was not significantly different from the 3D-printed arm phantom (*P*=.739), with 4% (3/68), 38% (26/69) and 57% (39/68), respectively, reporting that the training was inadequate, moderately adequate, and adequate.

### Comparison to Prior Work

Our results confirm the results of recently published studies [10,11] indicating the perceived realism of 3D-printed arm phantoms for practicing puncturing techniques is sufficient. Hyndman et al [10] printed a 3D-model of the upper limb vasculature to practice the Seldinger technique and assessed student satisfaction on 31 medical students. The use of the 3D-printed model improved anatomic understanding and application of the Seldinger technique of the students. Raffaela et al [11] developed a 3D-printed pediatric phantom to train ultrasound-guided placement of peripheral central venous catheters in children. The model was rated as highly realistic in terms of morphology and functionality for the overall simulation, by 20 expert specialists.

### Strengths and Limitations

One of the strengths of this study is that the characteristics of the two groups were comparable. Second, the questionnaire provided very valid suggestions for the improvement of adequacy of training on the next version of the 3D-printed arm, for example, a skin that endures more injections and could be easily replaced, a less heavy phantom to enable easy positioning of the arm, a skin that is thinner to resemble a real skin, and different stages of difficulty in the arm phantom, like thinner, smaller and rolling vessels. A third strength of this study is the fact that a 3D-printed arm phantom can lower the costs of training significantly and that the design can be accessible worldwide. The costs of the 3D-printed arm phantom (approximately US $80) are substantially lower compared with the commercially available arm phantom (approximately US $1100). A digital model for 3D-printing can be shared for free, like the 3D-simulator for arthroscopy given by Ferras et al [12] can be downloaded and printed for free in any 3D printer. Sritharan et al [13] are currently performing a review on how the use of open-source databases for the distribution of simulator designs used for 3D-printing can promote credible solutions for health care training while minimizing the risks of commercialization of designs for profit.

One of the limitations of the study is the potential training effect, as both randomized groups reported slightly higher perceived realism of the second training arm (although not statistically significant). Second, the limited sample size of 68 participants may reduce generalizability. A third limitation is the variability in participant experience, which could potentially influence perceptions of realism. Furthermore, the timeline of distribution into the two groups was not comparable (at the start, most participants started in group 1). As a result, the skin of particularly the 3D printed arm showed multiple injection sites and there was leakage of blood through these injection sites. This might be a reason why participants rated the arms as less realistic. As a result of these limitations, further research is recommended to validate our findings in a broader population. Ultimately, additional outcome measures, including success rates or skill retention, could be added.

### Future Directions

Our study shows that the 3D-printed arm phantom is comparable to the commercially available arm phantom in terms of perceived realism and in the possibility to offer an adequate training of the venipuncture. The advantage of 3D-printing is the possibility that it offers to design and print tailor-made arms. In a 3D-printed arm, different vascular morphologies can be designed like vascular morphologies that are palpable and superficially located or deep vascular morphologies that need ultrasound guidance to puncture instead. Moreover, vascular access with aneurysms can be designed to train adequate cannulation in these challenging scenarios. A discipline in which these challenging scenarios are very common is kidney dialysis. The Kidney Disease Outcomes Quality Initiative 2019 strongly recommends training and retraining hemodialysis staff to ensure the maintenance of competency of cannulation skills [14]. They encourage future research to evaluate whether training on cannulation simulation models improves cannulation competency and reduces cannulation complications. A 3D-printed tailor-made arm would offer a simulation model adjusted for this application, in line with the strong recommendation of the Kidney Disease Outcomes Quality Initiative.

An additional advantage of the 3D-printed arm over the commercially available arm is the possible improvements on the skin. The skin can be designed in such a way that it can be easily removed for proper cleaning of blood leakage from the punctured vessels. This was a time-consuming task with the commercially available arm. Also, a different material can be chosen that does not show the location of the previous punctures and thus forces the trainee to palpate the localization of the vessels.

With implementation of the 3-D printed arm phantom for venipuncture training, substantial amounts of health care cost can be saved; when offering adequate training opportunities for venipuncture, the number of contacting the anesthesiology department to perform the venipuncture procedure instead can be lowered.

### Conclusions

The 3D-printed arm phantom is as realistic and provides an equally adequate training compared to the commercially available arm phantom. However, the 3D-printed arm offers the possibility to design different models representing several levels of difficulty for vascular morphology. This potentially lowers the number of venipuncture failures by preparing health care workers on challenging scenarios.

## Supplementary material

10.2196/71139 Multimedia Appendix 1 Questionnaire
