# Correction: Comparing the Perceived Realism and Adequacy of Venipuncture Training on an in-House Developed 3D-Printed Arm With a Commercially Available Arm: Randomized, Single-Blind, Cross-Over Study

**DOI:** 10.2196/89670

**Published:** 2025-12-22

**Authors:** Susan Gijsbertje Brouwer de Koning, Amy Hofman, Sonja Gerber, Vera Lagerburg, Michelle van den Boorn

**Affiliations:** 1 3D Lab, Department of Computerization, Automation and Medical Technology (iMED), OLVG Hospital, 9 Oosterpark, Amsterdam, 1091 AC, The Netherlands, 31 020 599 91 11; 2 Department of Research and Epidemiology, OLVG Hospital, Amsterdam, The Netherlands; 3 Skillslab, OLVG Hospital, Amsterdam, The Netherlands; 4 Department of Medical Physics, OLVG Hospital, Amsterdam, The Netherlands; 5 Department of Medical Physics and Instrumentation, St. Antonius Ziekenhuis, Nieuwegein, The Netherlands

In “Comparing the Perceived Realism and Adequacy of Venipuncture Training on an in-House Developed 3D-Printed Arm With a Commercially Available Arm: Randomized, Single-Blind, Cross-Over Study” [1], the authors made one addition.

The following affiliation has been added as affiliation 5 and attached to author VL:

Department of Medical Physics and Instrumentation, St. Antonius Ziekenhuis, Nieuwegein, The Netherlands

The correction will appear in the online version of the paper on the JMIR Publications website, together with the publication of this correction notice. Because this was made after submission to PubMed, PubMed Central, and other full-text repositories, the corrected article has also been resubmitted to those repositories.
