# Supplementary material for: Comparing the Perceived Realism and Adequacy of Venipuncture Training on an in-House Developed 3D-Printed Arm With a Commercially Available Arm: Randomized, Single-Blind, Cross-Over Study
Source: JMIR Med Educ. 2025 Nov 4;11:e71139. doi: 10.2196/71139 (PMC12584987; doi:10.2196/71139)
Supplement: Multimedia Appendix 1 [file mededu-v11-e71139-s001.docx]

**Questionnaire 1**

1. You are a:

Nurse/Resident/Medical intern/Doctors’ assistant/Other:

1. What department are you currently working on:
2. I have … years of experience with venipuncture.
3. I qualify my venipuncture skills as

| Incompetent |  |  |  |  |  |  |  |  |  | Competent |
| --- | --- | --- | --- | --- | --- | --- | --- | --- | --- | --- |
| 0 | 1 | 2 | 3 | 4 | 5 | 6 | 7 | 8 | 9 | 10 |

1. How often do you need only one attempt for venipuncture?

| Never |  |  |  |  |  |  |  |  |  | Always |
| --- | --- | --- | --- | --- | --- | --- | --- | --- | --- | --- |
| 0% | 10 | 20 | 30 | 40 | 50 | 60 | 70 | 80 | 90 | 100% |

1. How often did you need to get help because you failed for successful venipuncture within two attempts, during last month?

… times

1. How would you prefer the training for venipuncture?

…

1. How were you trained for venipuncture before?

I am not trained/ using an injection arm phantom/ venipuncture on collegue/ other:

1. On which arm did you train today?

Arm A/Arm B

1. How realistic was the arm phantom?

| Not realistic |  |  |  |  |  |  |  |  |  | Realistic |
| --- | --- | --- | --- | --- | --- | --- | --- | --- | --- | --- |
| 0 | 1 | 2 | 3 | 4 | 5 | 6 | 7 | 8 | 9 | 10 |

1. How adequate was the training to practice the venipuncture?

Inadequate / moderately adequate / adequate

1. Do you have any suggestions/recommendations to improve the training?

…

**Questionnaire 2**

1. On which arm did you train today?

Arm A/Arm B

1. How realistic was the arm phantom?

| Not realistic |  |  |  |  |  |  |  |  |  | Realistic |
| --- | --- | --- | --- | --- | --- | --- | --- | --- | --- | --- |
| 0 | 1 | 2 | 3 | 4 | 5 | 6 | 7 | 8 | 9 | 10 |

1. How adequate was the training to practice the venipuncture?

Inadequate / moderately adequate / adequate

1. Do you have any suggestions/recommendations to improve the training?

…
